# Body composition and venison quality of farmed red deer (*Cervus elaphus*) hinds reared on grass, *papilionaceous* or mixed pasture paddocks

**DOI:** 10.5194/aab-62-227-2019

**Published:** 2019-04-29

**Authors:** János Nagy, András Szabó, Tamás Donkó, Julianna Bokor, Róbert Romvári, Imre Repa, Péter Horn, Hedvig Fébel

**Affiliations:** 1 Kaposvár University, Faculty of Agricultural and Environmental Sciences, Bőszénfa Game Management Landscape Center, 3. Malom str., Bőszénfa, 7475, Hungary; 2 Kaposvár University, of Agricultural and Environmental Sciences, “Mycotoxins in the Food Chain” Research Group, Guba S. u. 40, 7400, Kaposvár, Hungary; 3 Somogy County Moritz Kaposi Teaching Hospital, Dr. József Baka Diagnostical, Oncoradiological, Research and Educational Center, Guba S. u. 40, 7400, Kaposvár, Hungary; 4 Kaposvár University, Faculty of Agricultural and Environmental Sciences, Guba S. u. 40, Kaposvár, Hungary; 5 National Agricultural Research and Innovation Centre, Research Institute for Animal Breeding, Nutrition and Meat Science, Gesztenyés str. 1, 2053, Herceghalom, Hungary

## Abstract

Red deer (*Cervus elaphus*) hinds (n=3×10) of identical initial body weight (BW, ca. 68 kg) were reared on a monocotyledonous grass (G group), on a grass–*papilionaceous* (GP group) or on pure papilionaceous pasture each of 2 ha (P group) for 219 d. At the end of the experiment carcass tissue composition was assessed by means of computer tomography, slaughter value and meat quality were characterized and tissue – *longissimus thoracis et lumborum* (LTL), thigh and liver – samples were taken for fatty acid composition analysis. The primary aim was to assess nutrition-driven differences.

Hinds of group P provided higher final BW (101 kg vs. 90 and 91.9 kg in groups G and GP, respectively) and higher BW gain (32.6 kg during the total period vs. 22.4 and 22.1 kg). The carcass weight exceeded those of the other groups significantly (68.8 kg vs. 59.3 and 63.2 kg), while there was no difference among groups in the perirenal fat weight and red color tone ($a^{*}$) of the LTL. Groups G and P differed significantly in the LTL weight (highest in P), its dripping loss (lowest in G), lightness (L; highest in P) and yellow color tone ($b^{*}$).

In the thigh muscle, LTL and liver the highest proportion of fatty acid CLA9c11t was reached on the G pasture, and the same trend was true for docosahexaenoic acid (DHA , C22:6 n3) in the muscles. The n6 / n3 fatty acid ratio was the highest on the P pasture in the liver and both muscles. The liver incorporated the highest proportion of linoleic acid (C18:2 n6) and converted it rather effectively to arachidonic acid (C20:4 n6), coupled with the lowest α-linolenic acid presence.

In conclusion, concerning muscle mass production, group P proved to be the most advantageous pasture; meanwhile LTL meat quality factors (dripping loss, DHA proportion, pH, color) were more favorable on the G pasture.

## 1 Introduction

Red deer (*Cervus elaphus*) is the most important big game of Hungary due to its imposing meat; thus, farmed gamekeeping is quickly spreading. In Hungary, wild living red deer are exposed to drastically changing seasonal variations; the spring–summer diet consists of green plants and leaves of high nutritional value. To compensate for the winter, the long mid-summer (a strong burden in Hungary) and nutritionally rather challenging feed shortages, additional feeding is applied for farmed and even wild herds, with questionable efficacy.

Venison, possessing special quality characteristics, is further influenced by multiple factors such as season and nutrition (gender, reproductive stage and age). The concept of a seasonal component was first shown by French et al. (1956) and it was primarily attributed to the photoperiod and feed intake. Wiklund et al. (2010) provided evidence for marked seasonal variation in pH, water holding capacity and calpain activity in European red deer meat.

Nutrition (primarily the true protein digestibility and the energy uptake) is a basic factor influencing venison quality and production intensity. According to Huapeng et al. (1997), red deer adapt to seasonal forage quality changes to maintain a relatively constant crude protein intake level and need a minimum digestible energy intake of 153.5 kcal BW${}^{0.75}$ (kg) per day to reach positive nitrogen balance. Because of poor wintertime vegetation in Hungary, farmed deer are dominantly fed on fermented grass silage, prepared from their permanent paddock or pasture (Szabó et al., 2013); the raw material is harvested in the spring. Studies comparing the effects of starch rich grain vs. pasture-grass finishing diets have usually been performed in domestic meat-producing ruminants, but data on red deer are scarce. McCaughey and Cliplef (1996) demonstrated that while pasture-finished steers had lower yields and darker meat, there were no effects on tenderness, juiciness, flavor and overall consumer acceptability. Special attention must concern the protein digestion of red deer, which is strongly influenced by catechin polymers (Huapeng et al., 1997); but this factor is generally of minor importance on intensively handled pastures coupled to farmed rearing (Mitchell et al., 1977).

In deer rearing it is important to acknowledge the impact of feed type (pasture vs. grain) on the quantity and quality of harvested meat. Accordingly, this targeted study investigated effects of three pasture types (from a carbohydrate-rich to a high-protein type) and the full exclusion of grain addition on the total body composition, final body weight and some meat quality characteristics, not characterized in such a setting so far.

## 2 Material and methods

### 2.1 Animals and slaughter

The weaned red deer calves were penned in boxes for 20 individuals in each throughout the winter at the Bőszénfa Game Management Landscape Center, Kaposvár University (46${}^{\circ }$13${}^{\prime }$43.20${}^{\prime \prime }$ N, 17${}^{\circ }$50${}^{\prime }$47.84${}^{\prime \prime }$ E; Bőszénfa, Hungary). Altogether 66 red deer hinds were randomly allocated to 3 different pasture-covered paddocks (11 April 2015, each 2 ha, 22 animals per paddock). Animals were dewormed once during the entire study interval (June), with orally administered Albendanin 5 % suspension (Pharmatéka Inc., Hungary). Initial body weight (BW) of animals was around 68 kg (Table 3).

Animals had access to water and salt blocks during the grazing period. At the end of rearing deer were shot (16 November 2015) after hurdling and bled-out within ca. 2–3 min after shooting by cutting the jugular vein. Animals were transported on an open lorry into the game carcass handling unit (ca. 1 km) and hanged in a digital-scale-supported facility. The temperature of the dissection room was 4–6 ${}^{\circ }$C. Dissection and organ weight measurements (Table 3) were performed immediately post mortem. Samples of a randomly selected 10 hinds per group (total n=3×10=30) were analyzed. For fatty acid analysis, liver, *m. longissimus thoracis et lumborum* (thoracic part) and *m. gluteus* samples (selectively dissected out from the entire thigh) were taken and stored frozen (-70 ${}^{\circ }$C) until analysis. Tranquilizers before shooting were avoided since farmed deer venison served as a commercial product. The shooting was performed within ca. 10 min for the entire study cohort. The study was performed under the hunting licence of the Bőszénfa Deer Park, allowance no. 2/1364–2/2011 by the Somogy County Government Agency, Directorate of Agriculture.

### 2.2 Pastures

The monocotyledonous grass-based pasture (G) was dominantly composed of perennial ryegrass (*Lolium perenne*) and common meadow grass (*Poa pratensis*) (Table 1). The *papilionaceous* pasture (P) was dominantly based on alfalfa (*Medicago sativa ssp. varia*), red clover (*Trifolium pratense*) and white clover (*Trifolium repens*). The grass–papilionaceae (GP) pasture was a mixture of the two types mentioned above. Pasture coenological compositional sampling and analysis was performed with the Braun–Blanquet method (Podani, 2006). In each paddock there were two sampling events (initial and final, April and November, respectively) taken by same diagonal way on four locations for determination of chemical and fatty acid composition. Results on the coenology are summarized in Table 1.

**Table 1:** Feed coenological composition of the three experimental pastures.

Species	Covered percent
	of the area
Papilionaceous pasture	
Alfalfa (*Medicago sativa ssp. varia*)	5.1 %–25 %
White clover (*Trifolium repens*)	5.1 %–25 %
Red clover (*Trifolium pratense*)	5.1 %–25 %
Perennial ryegrass (*Lolium perenne*)	0.1 %–1 %
Soft brome (*Bromus mollis*)	0.1 %–1 %
Common meadow grass (*Poa pratensis*)	0.1 %–1 %
Meadow fescue (*Festuca pratensis*)	0.1 %–1 %
Field eryngo (*Eryngium campestre*)	0.1 %–1 %
Common dandelion (*Taraxacum officinale*)	0.1 %–1 %
Broad-leaved dock (*Rumex obtusifolius L.*)	0.1 %–1 %
Common yarrow (*Achillea millefolium*)	0.1 %–1 %
Creeping thistle (*Cirsium arvense*)	0.1 %–1 %
Grass – papilionaceous mixed pasture	
Alfalfa (*Medicago sativa ssp. varia*)	0.1 %–1 %
White clover (*Trifolium repens*)	0.1 %–1 %
Red clover (*Trifolium pratense*)	1.1 %–5 %
Perennial ryegrass (*Lolium perenne*)	5.1 %–25 %
Soft brome (*Bromus mollis*)	0.1 %–1 %
Common meadow grass (*Poa pratensis*)	25.1 %–50 %
Meadow fescue (*Festuca pratensis*)	0.1 %–1 %
Cock's-foot (*Dactylis glomerata*)	0.1 %–1 %
Tall fescue (*Festuca arundinacea*)	0.1 %–1 %
Field eryngo (*Eryngium campestre*)	0.1 %–1 %
Common dandelion (*Taraxacum officinale*)	0.1 %–1 %
Shepherd's-purse (*Capsella bursa-pastoris*)	0.1 %–1 %
Common yarrow (*Achillea millefolium*)	0.1 %–1 %
Field bindweed (*Convolvulus arvensis*)	0.1 %–1 %
Annual gypsophila (*Gypsophila muralis*)	0.1 %–1 %
Grass pasture	
White clover (*Trifolium repens*)	0.1 %–1 %
Red clover (*Trifolium pratense*)	0.1 %–1 %
Perennial ryegrass (*Lolium perenne*)	5.1 %–25 %
Soft brome (*Bromus mollis*)	1.1 %–5 %
Common meadow grass (*Poa pratensis*)	5.1 %–25 %
Meadow fescue (*Festuca pratensis*)	0.1 %–1 %
Cock's-foot (*Dactylis glomerata*)	0.1 %–1 %
Tall fescue (*Festuca arundinacea*)	1.1 %–5 %
Annual fleabane (*Erigeron annuus*)	0.1 %–1 %
Welted thistle (*Carduus acanthoides*)	0.1 %–1 %
Field bindweed (*Convolvulus arvensis*)	0.1 %–1 %

The coenological samples were pooled in April (n=4) and also those from November (n=4), and these homogenates underwent chemical compositional analysis. For fatty acid composition, only the four samples harvested in April were allocated, but were not pooled.

### 2.3 Analysis of chemical composition of pastures

The chemical composition (Wendee analysis) of the plant samples was determined by AOAC (2000) methods for moisture (930.15), crude protein (984.13), ether extract (920.39), fiber (978.10) and ash (942.05). Analyses of neutral detergent fiber (NDF) and acid detergent fiber (ADF) were performed according to Van Soest et al. (1991). The Ca content was determined by the flame atomic absorption spectrophotometry (method 968.08. AOAC, 2000) and total P content was measured by colorimetry using the molybdovanadate method (method 965.17. AOAC, 2000).

### 2.4 Meat quality analysis

The *longissimus thoracis et lumborum* (LTL) pH was measured postmortem by a Testo 205 precision handheld pH meter (Testo SE & Co. KGaA, Lenzkirch, Germany), with its built-in, temperature controlled electrode (Testo cat. no. 0650 2051). The cut surface color of the fresh LTL was determined by a Minolta ChromaMeter 300 (Minolta Corp., Tokyo, Japan) apparatus (aperture diameter: 11 mm; observer: 10${}^{\circ }$; angle: 90${}^{\circ }$; illuminant: D65), after a blooming time of 45 min and results were interpreted in the CIELAB coordinate system (Commission International De I'Eclairage, 1976). Dripping loss was determined by the method of Honikel (1998). To determine the cooking loss, samples (100 g) were closed into sealed polyethylene bags and were cooked according to Lawrie (2006). The exudate weight, as expressed in the percentage of the initial sample weight, was referred to as cooking loss.

### 2.5 Analysis of fatty acid profile of organs and feed

Animal tissue (and herbal material) samples were extracted according to Folch et al. (1957). Fatty acid methyl esters were extracted into 300 µL ultrapure n-hexane for gas chromatography and analyzed as described earlier (Szabó et al., 2007). Fatty acid results were expressed as weight percent of total fatty acid methyl esters.

### 2.6 Computer tomography analysis for total carcass composition

Computer tomography (CT) scanning was performed to analyze eviscerated body (carcass) composition, 24 h after the slaughter. CT scanning of the entire carcass was carried out with a Siemens Somatom Sensation Cardiac multislice CT scanner (Siemens AG, Erlangen, Germany). The segmentation of the fat, muscle and bone tissues of the whole carcasses was performed according to the former studies (Romvári et al., 2006) based on the X-ray density value on the Hounsfield scale (HU) using the following ranges: fat (-20 to -200), muscle (+20 to +200), bone (>200). The LTL and whole thigh muscle volumes were segmented out manually using BrainMOD software (Spisák et al., 2013). The volumetric results were expressed as tissue volume (cm${}^{3}$).

### 2.7 Statistical analysis of results

Initial and final body weight data, as well as body weight gain, were compared with paired samples t test since deer were individually marked by ear tags. Fatty acid compositional data of the three groups were compared with analysis of variance (Tukey's post hoc test). For the meat quality traits the general linear model (GLM) procedure was applied, with group as a fixed factor and slaughter weight as a covariant in the model. SPSS (2012) was used for the analyses.

## 3 Results

### 3.1 Nutrient composition and fatty acid profile of pastures

According to the proximate composition analysis, the crude protein content increased in a graded manner in the following order: G<GP<P (Table 2). An opposite order was found for the ether extract, crude fiber, and for the N free extract content. Calcium was ca. 2 times higher in the P diet as compared to the G, while the phosphorus content was less different (Table 2).

**Table 2:** The chemical and the fatty acid composition of the pastures.

Feed	Grass			Grass–Papilionaceae			Papilionaceae	
Weende analysis	April	November		April	November		April	November
Dry matter (%)	261.0	267.0		235.0	228.0		192.0	210.0
Crude protein (g kg${}^{-1}$ DM)	84.3	123.6		102.1	153.5		192.7	228.6
Ether extract (g kg${}^{-1}$ DM)	19.2	22.5		17.0	21.9		20.8	19.0
Crude fiber (g kg${}^{-1}$ DM)	218.4	194.8		217.0	179.8		187.5	152.4
Ash (g kg${}^{-1}$ DM)	68.9	82.4		76.6	83.3		93.7	90.5
N free extract (g kg${}^{-1}$ DM)	609.2	576.7		587.2	561.4		505.3	509.5
NDF (g kg${}^{-1}$ DM)	448.3	430.7		446.8	386.0		322.9	295.2
ADF (g kg${}^{-1}$ DM)	283.5	220.9		246.8	214.9		229.2	195.2
ADL (g kg${}^{-1}$ DM)	22.9	33.7		29.8	17.5		36.5	33.3
Hemicellulose (g kg${}^{-1}$ DM)	164.8	209.8		200.0	171.1		93.7	100.0
Ca (g kg${}^{-1}$ DM)	7.4	4.4		4.6	5.6		14.6	11.1
P (g kg${}^{-1}$ DM)	2.3	3.2		3.0	3.6		2.9	3.5
Fatty acid composition	fatty acid composition, weight % of total fatty acids							
	April (means of 4 ind. samples ± SD)							
C10:0	$0.82\pm 0.84^{b}$			$2.07\pm 2.71^{c}$			$0.26\pm 0.19^{a}$	
C12:0	0.28±0.18			0.31±0.27			0.26±0.21	
C14:0	0.51±0.29			0.58±0.42			0.47±0.28	
C15:0	0.21±0.15			0.21±0.16			0.26±0.00	
C16:0	17.7±4.21			17.0±6.84			16.5±1.43	
C16:1 n7	$0.32\pm 0.26^{b}$			$0.30\pm 0.21^{b}$			$0.14\pm 0.07^{a}$	
C17:0	0.32±0.28			0.33±0.31			0.40±0.16	
C18:0	2.60±1.44			4.36±4.05			2.58±0.78	
C18:1 n9	3.72±2.03			5.38±5.25			2.00±0.72	
C18:2 n6	16.3±4.38			16.7±6.45			20.6±0.47	
C18:3 n3	55.1±15.1			51.0±27.9			55.1±3.30	
C20:0	0.44±0.13			0.47±0.30			0.53±0.07	
C20:1 n9	0.05±0.06			0.09±0.04			0.06±0.05	
C20:3 n6	0.87±0.83			0.73±0.23			0.20±0.03	
C20:3 n3	$0.09\pm 0.02^{a}$			$0.09\pm 0.01^{a}$			$0.11\pm 0.02^{b}$	
C22:0	$0.64\pm 0.12^{a}$			$0.74\pm 0.43^{b}$			$0.57\pm 0.02^{a}$	
∑ saturated	23.5±7.64			26.1±15.50			21.8±3.11	
∑ monounsaturated	$4.09\pm 2.23^{\mathrm{ab}}$			$5.77\pm 5.50^{b}$			$2.20\pm 0.74^{a}$	
∑ polyunsaturated	72.3±9.93			68.1±20.96			75.9±3.90	
∑ n6	$17.2\pm 5.21^{a}$			$17.1\pm 6.97^{a}$			$20.7\pm 0.61^{b}$	
∑ n3	$55.1\pm 5.1^{b}$			$51.1\pm 2.80^{a}$			$55.2\pm 3.29^{b}$	
∑n6/∑n3	$0.34\pm 0.19^{a}$			$0.44\pm 0.37^{b}$			$0.37\pm 0.01^{b}$	
Unsaturation index	202.0±34.4			192.4±15.38			208.9±10.1	
Average chain length	17.5±0.18			17.5±0.37			17.6±0.08	

${}^{a,b}$ Different small uppercase indices mean significant (P<0.05) intergroup differences by ANOVA.

Though larger differences were present in the feed coenological composition (Table 1), the feed fatty acid composition was moderately different among pastures (Table 2). The GP pasture provided a rather high capric acid (C10:0) proportion, while G and GP showed higher palmitoleic (C16:1 n7) and slightly lower eicosatrienoic acid (C20:3 n3) proportions. The total monounsaturation was the highest in the GP diet, ca. 2.5-times higher than in the P one. The n6 PUFA (polyunsaturated fatty acid) proportion was the highest in the P diet, exceeding both the G and the GP; meanwhile the n3 PUFA proportion was rather high in all diets (>50 %) with the G and the GP over 55 %. The number of double bonds per 100 acyl chains (UI, unsaturation index) and the average FA (fatty acid) chain length was not different among the diets.

### 3.2 Growth performance and carcass parameters

Results on initial and final body weight, overall weight gain and carcass parameters are summarized in Table 3. Groups G and GP provided rather similar growth and slaughter weights, while those on the pure *papilionaceous* pasture (P) reached significantly higher final body weight (BW), higher carcass weight and showed higher BW gain. Meanwhile in all above-mentioned traits G and even GP groups were significantly different form P, in the case of the LTL mass (and volume) significance was only attained between groups G (lowest mass) and P (highest mass), GP possessing an intermediate position. The absolute liver weight was significantly the lowest in the G animals; the GP and the P groups were not different from each other, but both exceeded the G mean value. The relative liver weight was not different among the groups. In contrast, the dissected total perirenal fat weight was not different among the three groups (Table 3).

**Table 3:** Somatic and meat quality (LTL) traits of the deer groups.

Trait	Grass	Grass–Papilionaceae	Papilionaceae
Body weight, initial (kg)	67.6±3.24	69.8±3.71	68.4±4.35
Body weight, final (kg)	$90.0\pm 3.53^{a}$	$91.9\pm 4.53^{a}$	$101.0\pm 6.62^{b}$
Body weight gain (kg)	$22.4\pm 2.27^{a}$	$22.1\pm 3.84^{a}$	$32.6\pm 5.68^{b}$
Carcass weight (kg)	$59.3\pm 2.71^{a}$	$63.2\pm 3.59^{a}$	$68.8\pm 5.35^{b}$
*longissimus thoracis et lumborum* mass (kg)	$1.85\pm 0.18^{a}$	$1.96\pm 0.15^{\mathrm{ab}}$	$2.11\pm 0.35^{b}$
Total body fat volume (cm${}^{3}$)	$4856\pm 508.2^{a}$	$5904\pm 541.4^{b}$	$5959\pm 1314^{b}$
Total body muscle volume (cm${}^{3}$)	$28\;661\pm 1169^{a}$	$30\;434\pm 1999^{a}$	$32\;580\pm 2225^{b}$
*longissimus thoracis et lumborum* volume (cm${}^{3}{)}^{*}$	$2676\pm 180.2^{a}$	$2819\pm 315.4^{\mathrm{ab}}$	$3143\pm 395.7^{b}$
Thigh muscle total volume (cm${}^{3}$)	$12\;550\pm 884.8^{a}$	$13\;645\pm 1458^{\mathrm{ab}}$	$14\;536\pm 904.5^{b}$
Total body bone volume (cm${}^{3}$)	$2843\pm 229.8^{a}$	$2780\pm 300.1^{a}$	$3586\pm 356.3^{b}$
Liver weight (kg)	$1.38\pm 0.11^{a}$	$1.51\pm 0.15^{b}$	$1.54\pm 0.13^{b}$
Perirenal fat weight (kg)	0.14±0.07	0.14±0.04	0.17±0.09
pH24	$5.49\pm 0.02^{a}$	$5.55\pm 0.07^{b}$	$5.49\pm 0.04^{a}$
Dripping loss (%)	$2.95\pm 1.00^{a}$	$4.93\pm 1.47^{b}$	$4.31\pm 1.33^{\mathrm{ab}}$
Cooking loss (%)	$27.5\pm 0.70^{a}$	$27.6\pm 0.97^{a}$	$26.8\pm 4.63^{a}$
L (lightness)	$33.5\pm 1.68^{a}$	$35.0\pm 2.12^{\mathrm{ab}}$	$36.7\pm 2.56^{b}$
$a^{*}$	20.3±1.49	21.1±1.63	20.8±2.42
$b^{*}$	$4.78\pm 0.91^{a}$	$5.98\pm 2.11^{\mathrm{ab}}$	$6.54\pm 0.96^{b}$

${}^{*}$ Right and left sides together${}^{a,b}$ Different small uppercase indices mean significant (P<0.05) intergroup differences by ANOVA.

**Figure 1:**
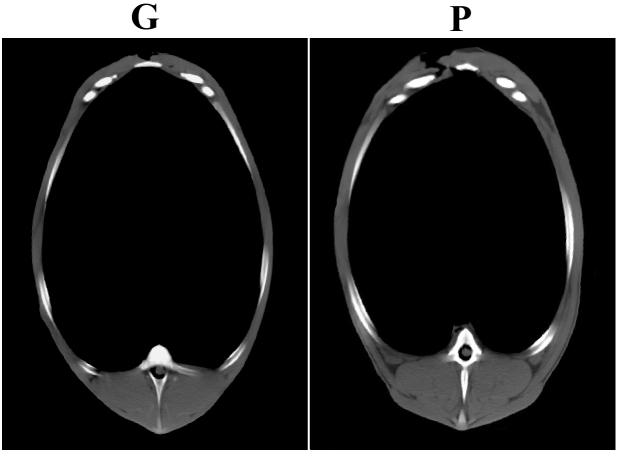
Cross sectional CT scan at identical anatomical location (at the joint of the sixth and seventh thoracal vertebrae) of two animals, one from group G (monocotyledonous grass pasture) and one from group P (pure *papilionaceous* pasture).

The computer tomographic scanning assessed the whole and eviscerated carcass composition, and results of this measurement are volumetric (cm${}^{3}$) (Table 3). The carcass total fat volume was significantly lower in group G, as compared to both others (GP and P), while the carcass muscle and bone volume of group P exceeded the others (G and GP) significantly. The volume of the LTL and thigh was significantly lower in group G as compared to group P, while group GP showed intermediate values. Figure 1 shows a cross sectional CT scan at an identical anatomical location (at the joint of the sixth and seventh thoracal vertebrae) of two animals, one from group G and one from group P, outlining the muscle and fat deposition differences.

### 3.3 Meat quality

The pH24 value of the G and P groups' LTL was identical and fell significantly below the value of the GP result. In contrast, the dripping loss of the GP groups exceeded that of the G group significantly, and cooking loss showed no intergroup differences. In the color components lightness (L) and yellow tone ($b^{*}$) were significantly higher in the P group, while the red tone ($a^{*}$) was identical in all groups (Table 3).

### 3.4 Fatty acid composition of LTL, thigh muscle and liver

The LTL showed higher conjugated linoleic acid (CLA9c11t) proportions in both grass fed groups (G and GP), and significantly lower proportions in the pure *papilionaceous* treatment (Table 4). In the saturated fatty acid (SFA) group arachidic acid (C20:0) provided lower proportions in group GP, as compared to the others (G and P). Docosahexaenoic acid (DHA, C22:6 n3) showed the lowest proportion in group P, the highest in group G, while group GP was not different from both. Tissue n6 PUFAs were not different among groups (only minor differences were found for eicosadienoic acid, C20:2 n6). In contrast, the n6 / n3 fatty acid ratio was significantly the highest in the P group, most likely as a result of the relatively high proportion of n6 dietary PUFAs of the P pasture.

**Table 4:** Fatty acid composition of the *m. longissimus thoracis et lumborum* of the three deer groups (n=10/group, means ± SD).

*longissimus thoracis et lumborum*	Grass	Grass–Papilionaceae	Papilionaceae
Fatty acid	Fatty acid composition, weight percent of total fatty acids		
C10:0	$0.02\pm 0.00^{a}$	$0.02\pm 0.00^{b}$	$0.03\pm 0.00^{\mathrm{ab}}$
C12:0	0.07±0.02	0.12±0.03	0.11±0.04
C14:0	$1.55\pm 0.49^{a}$	$2.77\pm 1.03^{b}$	$2.26\pm 1.34^{\mathrm{ab}}$
C15:0	0.39±0.08	0.45±0.10	0.44±0.16
C16:0	15.3±1.90	19.4±3.71	18.1±6.43
C16:1 n7	4.24±2.53	7.63±2.77	4.75±3.87
C17:0	0.82±0.12	0.79±0.17	0.79±0.26
C18:0	16.8±1.47	15.11±1.66	16.26±2.56
C18:1 n7	0.08±0.03	0.09±0.03	0.11±0.04
C18:1 n9	7.10±1.19	7.92±1.63	6.22±2.84
C18:2 n6	22.8±2.00	19.3±3.74	24.0±6.59
CLA9c11t	$0.29\pm 0.06^{b}$	$0.32\pm 0.07^{b}$	$0.18\pm 0.08^{a}$
CLA10t12c	0.18±0.13	0.11±0.09	0.16±0.09
C18:3 n6	0.10±0.03	0.09±0.03	0.11±0.04
C18:3 n3	5.86±0.75	5.52±1.52	4.82±0.99
C20:0	$0.13\pm 0.02^{b}$	$0.10\pm 0.02^{a}$	$0.14\pm 0.03^{b}$
C20:1 n9	0.03±0.02	0.03±0.01	0.03±0.01
C20:2 n6	$0.14\pm 0.01^{\mathrm{ab}}$	$0.13\pm 0.02^{a}$	$0.15\pm 0.03^{b}$
C20:3 n6	1.25±0.18	1.02±0.22	1.16±0.43
C20:3 n3	0.26±0.07	0.24±0.09	0.29±0.08
C20:4 n6	13.4±1.94	11.2±2.39	11.8±4.48
C20:5 n3	3.61±0.49	3.12±0.78	3.62±1.32
C22:0	0.15±0.11	0.08±0.06	0.15±0.10
C22:5 n3	4.41±0.46	3.58±0.56	3.64±1.15
C22:6 n3	$1.02\pm 0.17^{b}$	$0.87\pm 0.23^{\mathrm{ab}}$	$0.71\pm 0.27^{a}$
∑ saturated	35.3±2.63	38.8±4.60	38.3±8.81
∑ monounsaturated	11.4±3.29	15.7±4.16	11.1±6.46
∑ polyunsaturated	53.3±4.57	45.5±8.42	50.6±14.13
∑ n6	37.7±3.85	31.8±5.84	37.2±11.05
∑ n3	15.2±1.03	13.3±2.80	13.1±3.26
∑n6/∑n3	$2.48\pm 0.19^{a}$	$2.41\pm 0.26^{a}$	$2.81\pm 0.30^{b}$
Unsaturation index	176.5±12.29	156.6±22.32	163.6±38.9
Average chain length	18.1±0.16	17.8±0.25	17.9±0.43

${}^{a,b}$ Different small uppercase indices mean significant (P<0.05) intergroup differences by ANOVA.

In the thigh muscle basically similar results were found as in the LTL when concerning the CLA9c11t, DHA proportions and the n6 / n3 ratio (Table 5). Though not reflected in the total n3 proportion, α-linolenic acid (ALA, C18:3 n3) showed the highest proportion (ca. 7 %) in the GP group, differing significantly from group P.

**Table 5:** Fatty acid composition of the thigh muscle of the three deer groups (n=10 per group, means ± SD).

Thigh muscle	Grass	Grass–Papilionaceae	Papilionaceae
Fatty acid	Fatty acid composition, weight percent of total fatty acids		
C10:0	0.02±0.01	0.02±0.01	0.02±0.00
C12:0	$0.06\pm 0.03^{a}$	$0.07\pm 0.02^{\mathrm{ab}}$	$0.09\pm 0.02^{b}$
C14:0	1.42±0.86	1.68±0.53	2.00±0.68
C15:0	0.38±0.08	0.42±0.09	0.47±0.08
C16:0	14.3±2.25	15.4±2.03	16.8±3.47
C16:1 n7	4.89±2.84	5.04±2.32	4.87±1.79
C17:0	0.91±0.13	0.96±0.24	0.96±0.15
C18:0	17.2±1.46	17.8±2.23	17.0±0.91
C18:1 n7	$0.08\pm 0.03^{a}$	$0.10\pm 0.04^{\mathrm{ab}}$	$0.13\pm 0.05^{b}$
C18:1 n9	8.02±1.71	8.05±1.84	7.33±1.98
C18:2 n6	20.8±2.64	20.6±2.45	22.9±3.32
CLA9c11t	$0.35\pm 0.13^{b}$	$0.30\pm 0.07^{\mathrm{ab}}$	$0.22\pm 0.06^{a}$
CLA10t12c	0.04±0.01	0.04±0.03	0.04±0.01
C18:3 n6	0.09±0.07	0.08±0.02	0.10±0.02
C18:3 n3	$6.29\pm 0.75^{\mathrm{ab}}$	$6.94\pm 1.39^{b}$	$5.29\pm 0.79^{a}$
C20:0	$0.12\pm 0.02^{a}$	$0.14\pm 0.02^{\mathrm{ab}}$	$0.16\pm 0.03^{b}$
C20:1 n9	0.03±0.01	0.03±0.01	0.03±0.01
C20:2 n6	$0.15\pm 0.02^{a}$	$0.16\pm 0.01^{\mathrm{ab}}$	$0.17\pm 0.02^{b}$
C20:3 n6	1.21±0.23	1.12±0.23	1.22±0.32
C20:3 n3	$0.21\pm 0.03^{a}$	$0.22\pm 0.03^{\mathrm{ab}}$	$0.25\pm 0.04^{b}$
C20:4 n6	14.3±2.37	12.5±2.60	12.3±3.24
C20:5 n3	3.49±0.70	3.01±0.70	3.01±0.93
C22:0	0.04±0.02	0.05±0.01	0.05±0.01
C22:5 n3	4.68±0.90	4.33±0.61	4.01±0.97
C22:6 n3	$1.01\pm 0.17^{b}$	$0.96\pm 0.20^{b}$	$0.67\pm 0.25^{a}$
∑ saturated	34.4±3.08	36.5±2.78	37.6±4.20
∑ monounsaturated	13.0±3.97	13.2±3.25	12.3±3.45
∑ polyunsaturated	52.6±6.29	50.3±5.15	50.1±6.95
∑ n6	36.6±4.86	34.5±3.82	36.6±5.49
∑ n3	15.7±1.76	15.5±1.97	13.2±1.69
∑n6/∑n3	$2.33\pm 0.18^{a}$	$2.25\pm 0.28^{a}$	$2.77\pm 0.20^{b}$
Unsaturation index	183.2±18.3	173.1±16.4	167.6±22.5
Average chain length	18.2±0.22	18.1±0.15	18.0±0.23

${}^{a,b}$ Different small uppercase indices mean significant (P<0.05) intergroup differences by ANOVA.

In the liver, group G provided the highest oleic acid proportion (C18:1 n9), leading ultimately to the highest total monounsaturation of this group (Table 6). CLA9c11t showed the above-written muscle analogous distribution pattern also in the liver (G & GP > P). In the n6 PUFAs linoleic acid (C18:2 n6) proportion of group P exceeded groups G and GP, and P exceeded G as well in the arachidonic acid (C20:4 n6) proportion, leading ultimately to a dominance of n6 PUFAs in group P, inducing a shift towards the n6 PUFAs in the n6 / n3 ratio.

**Table 6:** Fatty acid composition of the liver of the three deer groups (n=10 per group, means ± SD).

Liver	Grass	Grass–Papilionaceae	Papilionaceae
Fatty acid	Fatty acid composition, weight percent of total fatty acids		
C10:0	0.02±0.01	n.d.	n.d.
C12:0	0.08±0.09	n.d.	n.d.
C14:0	0.85±0.60	0.73±0.13	0.75±0.12
C15:0	0.86±0.23	0.70±0.04	0.77±0.06
C16:0	14.4±3.09	13.6±1.70	13.0±0.77
C16:1 n7	1.03±0.45	0.87±0.21	0.99±0.19
C17:0	2.59±0.22	2.41±0.17	2.44±0.19
C18:0	32.0±2.33	31.9±2.10	32.1±0.71
C18:1 n7	0.16±0.05	0.12±0.02	0.14±0.02
C18:1 n9	$8.20\pm 1.70^{b}$	$6.30\pm 0.65^{a}$	$5.44\pm 0.82^{a}$
C18:2 n6	$5.99\pm 0.80^{a}$	$6.56\pm 0.66^{a}$	$8.09\pm 0.98^{b}$
CLA9c11t	$0.34\pm 0.09^{b}$	$0.25\pm 0.08^{a}$	$0.23\pm 0.04^{a}$
CLA10t12c	0.29±0.25	0.29±0.16	0.14±0.11
C18:3 n6	0.28±0.11	0.22±0.13	0.31±0.11
C18:3 n3	$2.25\pm 0.27^{a}$	$2.59\pm 0.45^{\mathrm{ab}}$	$3.05\pm 0.56^{b}$
C20:0	$0.13\pm 0.04^{b}$	$0.10\pm 0.02^{a}$	$0.11\pm 0.02^{\mathrm{ab}}$
C20:1 n9	0.13±0.07	0.14±0.02	0.09±0.05
C20:2 n6	0.32±0.26	0.30±0.11	0.29±0.08
C20:3 n6	1.51±0.34	1.59±0.28	1.69±0.45
C20:3 n3	$0.35\pm 0.09^{a}$	$0.44\pm 0.07^{a}$	$0.56\pm 0.10^{b}$
C20:4 n6	$12.2\pm 2.62^{a}$	$12.8\pm 0.82^{\mathrm{ab}}$	$14.1\pm 0.45^{b}$
C20:5 n3	3.28±1.16	3.61±0.74	2.86±0.62
C22:0	0.26±0.18	0.21±0.12	0.11±0.07
C22:5 n3	8.14±2.38	9.59±0.68	9.00±0.52
C22:6 n3	4.48±1.71	4.64±0.96	3.70±1.07
∑ saturated	51.1±5.68	49.7±1.18	49.3±1.01
∑ monounsaturated	$9.52\pm 2.11^{b}$	$7.43\pm 0.86^{a}$	$6.66\pm 0.90^{a}$
∑ polyunsaturated	39.4±7.40	42.8±1.08	44.1±1.32
∑ n6	$20.3\pm 3.456^{a}$	$21.44\pm 0.65^{a}$	$24.5\pm 1.27^{b}$
∑ n3	18.5±4.42	20.9±1.29	19.2±1.15
∑n6/∑n3	$1.13\pm 0.20^{\mathrm{ab}}$	$1.03\pm 0.08^{a}$	$1.28\pm 0.12^{b}$
Unsaturation index	169.2±31.6	181.7±6.10	179.0±5.83
Average chain length	18.5±0.31	18.6±0.06	18.5±0.06

${}^{a,b}$ Different small uppercase indices mean significant (P<0.05) intergroup differences by ANOVA.

## 4 Discussion

Modern consumers prefer healthy meat, of which one very good example is deer venison, being a characteristic red cut. According to Hoffman and Wiklund (2006), young female consumers prefer white and low-fat meat. However, there is an increasing trend in healthy human nutrition that is very well satisfied with red meat (beef, game, etc.) of optimal energetic (ca. 4.5 % fat content; Kay et al., 1981) and compositional properties. According to Wood et al. (2004), the optimal fatty acid profile of meat is characterized by a polyunsaturated to saturated FA ratio of 0.1 and an n6 / n3 FA ratio not more than 4. This is generally true for ruminants when reared on pastures.

### 4.1 Growth performance and carcass parameters

According to Daszkiewicz et al. (2015), farm-raised and wild fallow deer (*Dama dama*) produce meat of strongly different quality. Authors reported lower meat energy concentration, the farmed animals producing the poorer cuts, most probably due to the limited locomotor activity. In our study all animals were farmed so as to attain maximal population homogeneity, to exclude extra feed sources from outside the paddocks and to have a clear view on the effect of the pastures.

The marked surplus difference in muscle mass (and volume) of the P group (Fig. 1 and Table 3) was with the highest probability attributable to the higher (ca. two-fold during the entire period) crude protein intake. However, overall somatic growth is also dependent on skeletal development, which was partly ensured by the higher Ca content of the P group's diet (Table 2). An additive role of dietary Ca is that its higher intake induces an increase in m-calpain (requiring Ca for its activity) augmenting myoblast differentiation (Kwak et al., 1993). The increase in m-calpain correlates with the elevated cleavage of filamin, which occurs during the fusion process, suggesting that m-calpain plays an important role in the cytoskeletal reorganization taking place during myoblast fusion (Cronjé and Boomker, 2000). However, similar forced raising of deer hinds on pure *papilionaceous* pastures and farm conditions with lower motoric activity has only been reported in our earlier study (Szabó et al., 2013). It must be added that this is yet a less frequent, but possibly a spreading feeding/rearing condition, which proved to be rather effective in skeletal muscle mass production. Anyhow, alfalfa and clover, i.e., the P diet was poorer in dry matter and crude fiber and rather rich in protein, induce a quicker passage and lead ultimately to nutrient losses (Christie, 1982).

Behind the rich nutrient supply it is very interesting that even the dietary fatty acid profile enables profound muscle hypertrophy, since the P group took up more linoleic acid (C18:2 n6) from the pasture (Table 2), which was also reflected in the liver (Table 6). Linoleic acid – besides growth factors – has been reported to stimulate myogenic differentiation (Allen et al., 1985).

During the postnatal phase the muscle mass increase is due to hypertrophy (not hyperplasia), since muscle fiber number does not increase significantly after birth anymore (Cronjé and Boomker, 2000). Muscle DNA concentration continues to increase throughout the growing/raising period due to satellite cell proliferation, differentiation and fusion with the already existing muscle fibers. This is accompanied by increased cellular protein deposition when this nutrient is available in excess (Table 2, P group). The cessation of DNA accretion happens immediately when the animal approaches its mature size and precedes the decline in protein accretion. Muscle protein accretion is the net balance between the relative rates of muscle protein synthesis (i.e., gene transcription and translation into protein) and degradation (e.g., proteolysis), and changes in either can result in increased muscle mass (Cronjé and Boomker, 2000). Our results provide evidence that high-protein supply from the pasture can induce drastically differing growth and muscularity (Table 3, Fig. 1) in a phenotypically strongly homogenous population. Protein supply was with the highest probability over the demands, as underscored by the CT compositional results, namely the total body fat content (volume) was higher in the GP and the P groups. However, the total body muscle volume (as well as the carcass weight) showed a graded increase according to the increasing dietary crude protein level, reaching significant excess in the P animals. The abovementioned processes are indeed underscored by Asher et al. (2011), namely that feed energy and protein content are the nutritional drivers of calf growth in farmed red deer. (It is interesting that perirenal fat has been found as an indicator of deer body condition (Watkins et al., 1991), but we were unable to provide supportive data.)

### 4.2 Meat quality

Farm conditions significantly differ from the wild environment; from a production-oriented point of view this type of gamekeeping is spreading (Hoffman and Wiklund, 2006). According to Malmfors and Wiklund (1996), the meat quality parameters are definitely influenced by the nutritional status and physical condition of the deer, e.g., by the muscle glycogen content.

Daszkiewicz et al. (2015) measured ultimate pH values in wild and farm-raised fallow deer bucks below 6, the wild population providing the lower values. In our study the GP group showed higher values, but the difference was minor, though significant (see Table 3). Our results are rather similar to those published by Pollard et al. (2002) for red deer muscles (three cuts), pH ranging from 5.54 to 5.64 in the LTL. Our treatments induced minor and partly expected results. Since pH development is primarily depending on the glycogen-originated lactate production, there are two conditions leading to low pH: first, higher muscle glycogene content, characteristic for grazing animals on the grass pastures when already producing germs (hepatic glyconeogenesis from propionic acid), and as second, large muscle mass (P group) tending to have a higher amount of fast-twitch glycolytic (oxidative) fibers (Curry et al., 2012). This latter condition, namely a shift towards a lighter (L↑) muscle color, reached significance in the P animals, but provided an intermediate value already in the GP group. Interestingly, the $b^{*}$ color component provided the same alteration, meaning a shift on the blue to yellow axis; meanwhile $a^{*}$, referring to the green to red transition, was not significantly different among the groups.

The same trend was published by Triumf et al. (2012) for the L value, which has also been clearly confirmed in our study, namely larger muscle mass at the same age was coupled with increased lightness. Interestingly, in the P groups' samples this intensive hypertrophy did not lead to augmented dripping or cooking loss, nor to a more rapid postmortem pH fall. This is sound with the findings of Asher et al. (2011), suggesting that the optimal pasture for calves is the ryegrass/clover combination for growth to show their maximum genetic potential (and lactation capacity of dams).

Summarizing the meat quality factors, it was assumed that the pure *papilionaceous* pasture was able to augment overall somatic growth and LTL hypertrophy of red deer hinds, but is not more effective than the grass–*papilionaceous* pasture. The P group was only unique in providing lighter and less yellow LTL meat; meanwhile the GP treatment already increased dripping loss and pH24 as compared to the G group.

### 4.3 Fatty acid composition

The fatty acid composition in the case of game is generally limited to the analysis of the LTL, while we intended to describe the incorporation (and possible modification) of pasture-originated fatty acids into further edible tissues as well.

#### 4.3.1 Fatty acid profile of the pastures

Besides similar ether extract concentration, the fatty acid profile of the three pastures was moderately different (Table 2). From a nutritional point of view, the level of monounsaturated fatty acid (MUFA; being the highest in the GP group), the rather high total n6 PUFA proportion (P) and the uncommonly high n3 PUFA (G and P) proportions were the most decisive characteristics. Since in the rumen unsaturated FAs are fully or partially hydrogenated (if present in an unprotected form, e.g., free vegetable oil), or remnant double bonds are converted to *trans*- allocation or shifted to result in a conjugated series (Cheeke and Dierenfeld, 2010), there is a low importance to handle dietary FAs as a direct basis for ultimate tissue FA profile in ruminants.

#### 4.3.2 *m. longissimus thoracis et lumborum*

Although this muscle pair is of relatively low weight in the *cervidae*, there is the highest number of literature data on its FA profile. According to Hoffman and Wiklund (2006), the LTL of the most important ruminant game animals is characterized by ca. 35 %–40 % SFA, 15 %–20 % MUFA and 32 %–42 % PUFA (only in males). In contrast, when Daszkiewicz et al. (2015) compared farm-raised and wild fallow deer LTL FA profiles, they found lower MUFA (22 % vs. 29 %) and higher SFA (61 % vs. 55 %) in the farmed animals.

According to Quaresma et al. (2012), Iberian red deer (*C. elaphus hispanicus*) tenderloin FA composition is not gender-dependent and contains 43 % SFA, 25 % MUFA, 23 % n6 PUFA and ca. 7 % n3 PUFA with a n6 / n3 ratio of ca. 3.5.

In contrast, in this hind population, the level of FA saturation and that of monounsaturation was lower; meanwhile the PUFA level was uncommonly high (45 %–53 %), both in the case of the n6 (32 %–37 %) and especially by the n3 PUFAs (13 %–15 %). This ultimately led to a rather low n6 / n3 ratio, reaching its minimum in the GP animals (2.41, Table 4).

According to Quaersma et al. (2012), deer meat is originally poor in intramuscular fat, which is thus mostly composed of structural lipids, in particular membrane phospholipids (Okabe et al., 2002), under strong compositional control. There are two major conditions to point out in the present study. The first is the very high n3 PUFA proportions of the pasture, which ultimately led to uncommonly high muscular n3 PUFA proportions contributing to a remarkably high DHA (C22:6 n3) proportion, in particular in the G group. This exceeds the value published for Iberian red deer (0.41–0.43; Quaresma et al., 2012) and New Zealand red deer (0.13 %–0.15 %; Triumf et al., 2012). Though PUFA are generally toxic compounds towards the ruminal microflora, we keep the assumption that the feeding methods applied were still effective in increasing the n3 and the PUFA proportions of the intramuscular fat. It needs to be added that the rumen is strongly reductive, thus linoleic acid (LA) (more abundant in P, see Table 2) may partially overcome the bio-hydrogenation due to its “protection”, namely its presence in the chloroplasts (likewise “caging”). These organelles are generally engulfed by rumen protozoa, and are thus acting as a source of “protected fatty acids” (Huws et al., 2009).

It has also to be added that G and GP groups showed higher CLA9c11t proportion in the LTL FA profile. This may refer to a higher LA to CLA conversion in both of these groups.

It has also to be added that the paddocks were fenced and the farmed hinds were kept merely on the pastures (bush and leaves were not available); meanwhile leaves can contribute up to 40 %–45 % to the red deer diet (Trdan et al., 2003).

Another process to mention is the effective formation of CLA9c11t and rumenic acid, which was most characteristic in the G and GP animals. According to Cheeke and Dierenfeld (2010), the first step of linoleic acid ruminal modification is the formation of rumenic acid, followed by the formation of *trans*-vaccenic acid and finally by full reduction to stearic acid (C18:0). According to Lanza et al. (2015), rumenic acid production is thus dependent on LA supply and the activity of delta-9 desaturation.

In our study it was rather interesting that even the n6 FA precursor, LA augmented the accretion of tissue arachidonic acid (C20:4 n6), which was most pronounced in the P animals. In contrast, high LA intake was found to not exert such an effect in growing lambs, nor augmenting the synthesis (and tissue incorporation) of C20:2 n6 and C20:3 n6 (Christie, 1981). The reason for this is that dietary intake and retention of essential FAs is very effective in ruminants, while elongation and further polydesaturation processes are energy demanding, thus being predominant in animals of high energy intake or intensive growth (Christie, 1981).

#### 4.3.3 Thigh FA composition

As compared to the LTL, the thigh muscle provided similar reactions towards the divergent feeding regimes. Fatty acids showing marked proportional alterations were CLA9c11t, ALA, arachidic acid (C20:0), eicosadienoic acid (C20:2 n6) and eicosatrienoic acids (C20:3 n6), as well as DHA. The alteration of the n3 FAs, in particular the proportional increase in ALA in the GP group, is of special interest, since its tissue incorporation seems to happen on a bypass pathway fully avoiding bacterial, and only partly entering protozoa metabolism (Christie, 1981; Huws et al., 2009). A similar controversy is the fact that the GP pasture was less rich in ALA, while the animals on this pasture provided the highest intramuscular fat proportion of this acid. ALA is generally cytotoxic towards rumen microbiota, and is mostly undergoing immediate ruminal biohydrogenation (to trans-monoenoic acids or even stearic acid); most probably the moiety present in plant phosphatides and chloroplasts was avoiding biodegradation. Morimoto et al. (2005) reported that ruminant somatic cells express desaturases (Δ5 and Δ6) to counterbalance the saturating effect of the rumen microbial biohydrogenation. This may be indeed the reason for the rather high n3 PUFA presence even in the polydesaturated cases like DHA.

Altogether, the thigh muscle showed a FA composition which was strongly similar to the LTL and is sound with the human dietetic recommendations (Wood et al., 2004).

#### 4.3.4 Liver FA profile

Though hepatic lipid metabolism in ruminants is extraordinary complex in terms of lipid fractions, in the frame of the present approach liver was handled as an edible tissue and its total FA composition was assessed as depending on the pasture fatty acid profile.

The basic difference, as compared to muscles was the graded decrease in oleic acid in parallel with the inclusion of *papilionaceae*, and also the significant, but opposite, alteration of linoleic acid. Moreover, ALA proportion was lower, as compared to the muscles; meanwhile DHA showed a specific hepatic recruitment, without intergroup differences.

The ALA to DHA conversion is rather poor in the mammals and it has been shown that rumen bacteria are capable of even for de novo DHA synthesis, primarily on grass feeding (Scollan et al., 2006). This assumption is not only underscored by the DHA absence in the diet, but as well by the enrichment of the n3 PUFA precursor of DHA, docosapentaenoic acid. According to the results, the G pasture seems to enrich red deer liver in DHA and effectively down regulate the entire metabolism of n6 PUFAs, as proven by the low LA proportion, and consequently the same significant trend was found for arachidonic acid.

## 5 Conclusion

Rearing red deer hinds on three different pasture paddocks led to different growth, fat deposition and muscle hypertrophy intensity. Pure *papilionaceous* diet led to the most expressed muscle building and fat deposition, but affected the LTL color to a more yellow and brighter tone. The fatty acid profile of the LTL and the thigh muscles was modified by the pastures in a detectable manner. Intensive growth was coupled with lower n3 FA proportions (ALA, DHA), and increased the n6 / n3 FA ratio (P pasture). Muscle dripping loss was the lowest and DHA proportion was the highest on the G pasture. Liver was enriched in LA with increasing *papilionaceous* ratio, while monounsaturation decreased in parallel. Carcass fat content was increased in parallel with growth intensity.

In summary, when comparing the pastures, skeletal muscle mass production was the most effective factor on the P variant; in contrast, LTL meat quality characteristics (dripping loss, DHA proportion, pH, color) were more advantageous on the G pasture.

## Data Availability

Research data are fully available on request from the corresponding author.
